# Geographical coverage of SARS-CoV-2 screening and care centers in Haiti: what do national surveillance data tell us?

**DOI:** 10.1186/s12889-024-19262-7

**Published:** 2024-06-28

**Authors:** Marcmy Presume, Jean Gaudart, Edwige Michel, Samson Marseille, Mathias Altmann

**Affiliations:** 1 Directorate of Epidemiology Laboratories and Research (DELR), Ministry of Public Health and Population (MSPP), Research Institute for Sustainable Development (IRD), Bordeaux Population Health Research Centre, EMR 271, Bordeaux, France; 2 Aix Marseille Univ, INSERM, IRD, SESSTIM, ISSPAM, AP-HM, Hospital La Timone, BioSTIC Unit, Marseille, France; 3 Directorate of Epidemiology Laboratories and Research (DELR), Ministry of Public Health and Population (MSPP), Port-Au-Prince, Haiti; 4 University of Bordeaux, National Institute for Health and Medical Research (INSERM) UMR 1219, Research Institute for Sustainable Development (IRD) EMR 271, Bordeaux Population Health Research Centre, Bordeaux, France

**Keywords:** COVID-19, SARS-CoV-2, Haiti, Spatio-temporal analysis, Geo-epidemiology

## Abstract

**Background:**

In Haiti, reported incidence and mortality rates for COVID-19 were lower than expected. We aimed to analyze factors at communal and individual level that might lead to an underestimation of the true burden of the COVID-19 epidemic in Haiti during its first two years.

**Methods:**

We analyzed national COVID-19 surveillance data from March 2020 to December 2021, to describe the epidemic using cluster detection, time series, and cartographic approach. We performed multivariate Quasi-Poisson regression models to determine socioeconomic factors associated with incidence and mortality. We performed a mixed-effect logistic regression model to determine individual factors associated with the infection.

**Results:**

Among the 140 communes of Haiti, 57 (40.7%) had a COVID-19 screening center, and the incidence was six times higher in these than in those without. Only 22 (15.7%) communes had a COVID-19 care center, and the mortality was five times higher in these than in those without. All the richest communes had a COVID-19 screening center while only 30.8% of the poorest had one. And 75% of the richest communes had a COVID-19 care center while only 15.4% of the poorest had one. Having more than three healthcare workers per 1000 population in the commune was positively associated with the incidence (SIR: 3.31; IC95%: 2.50, 3.93) and the mortality (SMR: 2.73; IC95%: 2.03, 3.66). At the individual level, male gender (adjusted OR: 1.11; IC95%: 1.01, 1.22), age with a progressive increase of the risk compared to youngers, and having Haitian nationality only (adjusted OR:2.07; IC95%: 1.53, 2.82) were associated with the infection.

**Conclusions:**

This study highlights the weakness of SARS-CoV-2 screening and care system in Haiti, particularly in the poorest communes, suggesting that the number of COVID-19 cases and deaths were probably greatly underestimated.

**Supplementary Information:**

The online version contains supplementary material available at 10.1186/s12889-024-19262-7.

## Background

The COVID-19 pandemic was the first health disaster that led to the confinement of more than 3 billion people [1]. With a basic reproduction number estimated at 2.2 in January 2020, Its speed of diffusion was impressive [2]. Between January 1, 2020 and December 31, 2021, the World Health Organization (WHO) estimated at 14.9 million the total number of deaths, directly or indirectly associated with the COVID-19 pandemic, with a concentration in Southeast Asia, Europe and the Americas [3].

One of the major challenges in controlling epidemics with person-to-person transmission is to be able to quickly detect cases to isolate them and interrupt the chain of transmission. For COVID-19, one of the greatest difficulties in case detection was the high proportion of asymptomatic but contagious forms [4, 5]. Since the severity of the disease was largely linked to age with younger people more likely to be asymptomatic, [4] it was particularly complicated to detect all cases in countries where nearly half of the population is under the age of 18. A previous study conducted in Haiti showed that 32% of COVID-19 cases diagnosed on an outpatient basis from March 18 to August 4, 2020 were asymptomatic and that 75.6% of symptomatic cases were mild [6].

Studies have shown that there was an even greater underestimation of cases when resources are limited at the national level. In the Dominican Republic, a population seroprevalence study was conducted from June to October 2021 and revealed that 77.5% of the population had previously been infected with the SARS-CoV-2, while the official reported cumulative incidence was less than 32 cases per 1000 population [7]. In the WHO African region, a study modeling the COVID-19 pandemic in 47 countries based on data from January 2020 to December 2021 estimated that less than 1.4% of cases were reported [8].

In Haiti, at the very beginning of the epidemic, the Multisectoral Commission for the Management of COVID-19 predicted that the country was going to have millions of COVID-19 cases in the best scenario, to reach up to eight million in the worst one with hundreds of thousands hospitalized and thousands deaths [9]. On December 31, 2021, the country cumulated only 26 225 COVID-19 reported cases and 773 related deaths [10], well below forecasts and suggesting that Haiti had been spared the pandemic. Haiti was one of the countries with the lowest COVID-19 vaccination coverage in the world, with 1.1% of the population having received at least their first dose and 0.7% of the population being fully vaccinated [10].

Little is known about the real impact of the epidemic in Haiti, with exception of two seroprevalence studies carried out in health centers among patients already attending these hospitals [11, 12]. They both agreed that the number of cases in Haiti was underestimated. Through our study, we analyzed factors at communal and individual level that might lead to an underestimation of the true burden of the COVID-19 epidemic in Haiti during its first two years, by: a) performing temporal and spatial description of the epidemic; b) determining socioeconomic factors associated with the distribution of the epidemic at the commune level; c) and determining individual risk factors associated with the infection.

## Methods

### Data source

This study was conducted in Haiti, using national surveillance data from March 2020 to December 2021, and other secondary data. The entire healthcare system was not involved in the fight against the epidemic. The ministry of health has designated and renovated specific centers throughout the country to receive patients for COVID-19 screening and care. COVID-19 screening centers were used to receive people for testing purpose, and COVID-19 care centers for COVID-19 patients requiring hospitalization. In addition to classic reports, individual data were also reported using an investigation form, regardless of COVID-19 test results. A detailed description of the surveillance system was provided by Lucien et al. [6].

Individual data included symptomatic people who performed a SARS-CoV-2 antigen or polymerase chain reaction (PCR) test during the study period. The variables considered are the following: Demographic variables (gender, age group, department of residence [administrative level higher than the commune], having Haitian nationality only [a proxy of low socioeconomic conditions in Haiti], Healthcare worker); Comorbidities (having at least one comorbidity); Lifestyle (tobacco and alcohol consumption); and result of the SARS-CoV-2 test. Aggregated data related to COVID-19 were from official reports of the ministry of health [13]. The report as of December 31, 2021 was used to extract the number of cumulative cases and deaths aggregated by commune of residence, age group and gender. The various daily reports published during the study period were used to extract new cases, deaths and number of tests. COVID-19 cases were based on lab test results (SARS-CoV-2 antigen or PCR). COVID-19 death was defined as death of a confirmed case or postmortem confirmation of COVID-19, unless there was another obvious cause of death unrelated to COVID-19 disease (such as trauma) [13]. Population figures were extracted from the last estimation of the population in 2015 by the Haitian Institute of Statistics and Informatics (IHSI) [14]. They were used to estimate the urbanization rate, the household density, the proportion of men, and the proportion of adults (those aged 18 and over) per commune. The number of healthcare centers and the number of healthcare worker per population were extracted from the most recent report on the Assessment of Health Care Service Delivery 2017–2018 (EPSSS 2017 -2018) [15]. The average distance from the centroid of the commune to the nearest COVID-19 screening and care center was calculated considering the official list of COVID-19 screening and care centers published on the website of the ministry of health and the road network. The altitude was calculated using a 20-m contour model. Geospatial data such as shapefiles of administrative boundaries, contours, and the road network to generate other variables such as the altitude level of communes, and others came from the National Center for Geospatial Information (CNIGS). Data used to calculate the proportion of the population in need of humanitarian intervention were from an estimation by the United Nations Office for the Coordination of Humanitarian Affairs (OCHA), in collaboration with Humanitarian Partners for the year of 2021 [16, 17]. People in need of humanitarian intervention were identified by combining information from the three sub-pillars of humanitarian conditions: living conditions, survival and well-being [18]. Data related to the number of COVID-19 cases and deaths by age group and gender, and the number of tests performed during the first 653 days of the epidemic in Dominican Republic, France and United States were from the WHO reports [19]. The estimated population data in 2020 used for the estimation of the standardized cumulative incidence, and mortality was extracted from PopulationPyramid.net [20]. Data related to the different SARS-CoV-2 variants in Haiti were extracted from the GISAID platform [21].

### Statistical analysis

Second, we described the epidemic in Haiti by performing a temporal description of the confirmed and reported cases and deaths. Time series of daily number of performed tests and positivity rate, and daily cases and deaths smoothed over seven days and superimposed on the SARS-CoV-2 variants curve was produced. Since we did not have standard indexes to classify commune’s poverty level, we applied the natural break of JENK on the proportion of the population in need of humanitarian intervention to classify the communes, creating classes in a way that best grouped similar values together and maximized the differences between classes [22]. We classified the communes of residence according to three levels of poverty: “high”, “moderate” or “low” if the proportion of the population in need of humanitarian intervention was greater than 46%, between 32 and 46%, and less than 32%, respectively. We performed a spatial description of the reported confirmed cases and deaths stratified by the accessibility to screening and care centers, and the poverty level of the commune. We designed maps and for their representation, we used the natural break of JENK as a choice of discretization. The Kernel Density Estimation method was used to design the heat map [23]. We performed the Kulldorff's spatial scanning method to detect clusters for COVID-19 cases and deaths [24]. In order to avoid edge effect, the maximum cluster size was set at a circle with a radius of 60 km.

Third, in order to determine socioeconomic factors associated with the incidence and the mortality, we performed a spatial regression analysis by taking the commune of residence as the spatial unit. Cumulative incidence, and mortality were used as dependent variables. We performed multivariate Quasi-Poisson regressions adjusted on socioeconomic covariates, using Generalized Additive Model (GAM). Log(population) was used as an offset to estimate standardized incidence ratios (Eq. 1), and standardized mortality ratios (Eq. 2). S(lon,lat), representing a Gaussian kriging smoother based on the geographical coordinates of each commune centroid, was used to take spatial autocorrelation into account (Eqs. 1 and 2).

1 $$\log\left(\mathrm{cases}\right)\;=\mathrm{factors}\;+\;\mathrm{offset}\left(\log\left(\mathrm{population}\right)\right)\;+\;\mathrm s\left(\mathrm{lon},\mathrm{lat}\right)\;\sim\;\mathrm{QuasiPoission}$$

2 $$\log\left(\mathrm{deaths}\right)\;=\mathrm{factors}\;+\;\mathrm{offset}\left(\log\left(\mathrm{population}\right)\right)\;+\;\mathrm s\left(\mathrm{lon},\mathrm{lat}\right)\;\sim\;\mathrm{QuasiPoission}$$

Finally, in order to determine individual factors associated with the infection, we performed individual analysis of being diagnosed positive for SARS-CoV-2, defined by SARS-CoV-2 PCR or antigen test result. Sample description, and bivariate analysis were performed using logistic regression. We performed multivariate analysis using mixed-effects logistic regression model and including the department of residence as random effect.

Additionally, we provided in the appendix a comparison of the incidence and mortality in Haiti with other countries, assessing if the country reported less cases (Additional file 1). We estimated standardized cumulative incidence and mortality on 653rd day after the first official reported case in each country, using the direct standardization method, standardizing on age and gender, and taking the world population as a reference. Estimations were made for Haiti, Dominican Republic, France, and United States for comparison purposes.

We performed sensitivity analysis for the produced models, using different simulation methods to assess their robustness and the stability of the results (Additional file 2).

We used MS Excel 2019 for data processing, R version 4.2.2 with the mgcv and lme4 packages for data analysis, SaTScan v10.1 for cluster detection, and QGIS version 3.26 for data extraction and production, and for cartography.

## Results

### Temporal description of the epidemic in Haiti

**Fig. 1:**
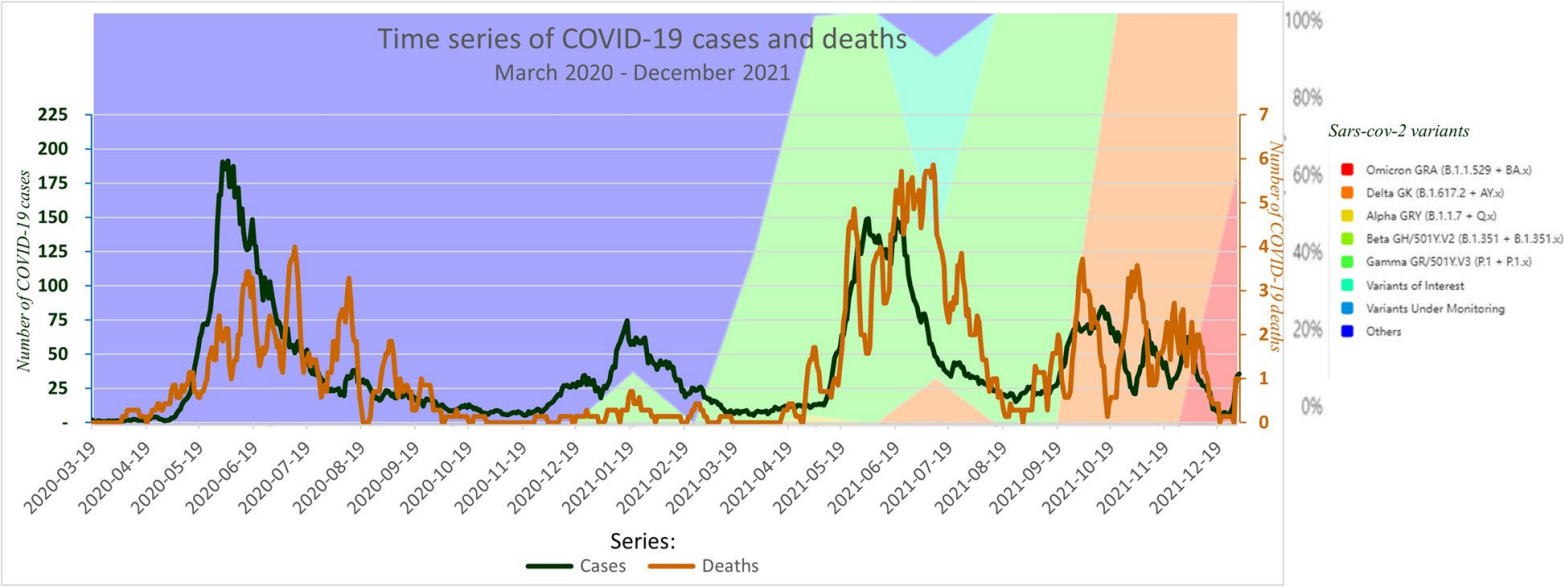
Time series of COVID-19 incidence and mortality, and SARS-CoV-2 variants, Haiti, March 2020-December2021. In dark green, the COVID-19 cases curve linked to the absolute values on the primary axis (on the left). In brown, the COVID-19 related deaths curve linked to the absolute values on the secondary axis (on the right) in red. The background colors in red correspond to the Sars-CoV-2 variants defined according to the legend on the right and linked to the values in percentage on the tertiary axis (on the right) in gray

**Fig. 2:**
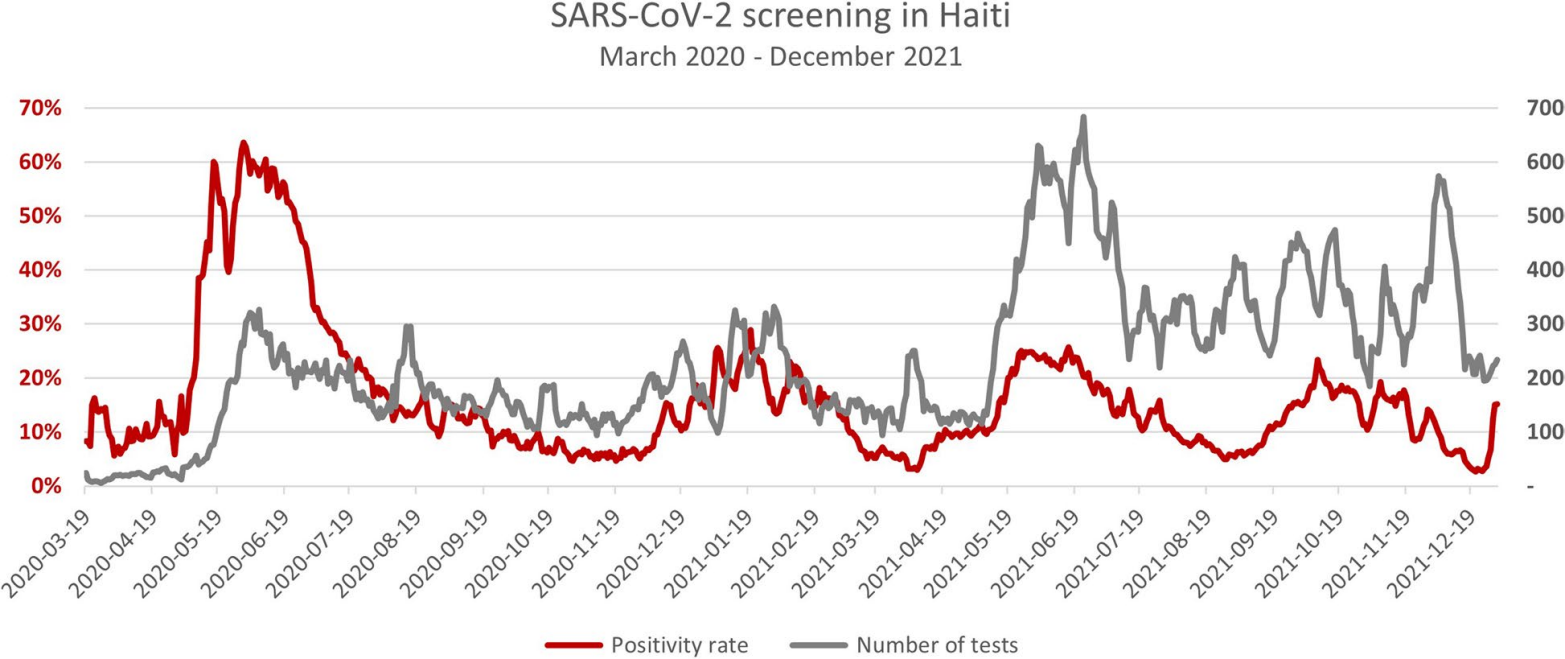
Time series of the positivity rate and Sars-CoV-2 tests number, Haiti, March 2020-December2021. In dark red, the positivity rate curve linked with the values in percentage on the primary axis (on the left). In gray, the number of test curve linked with the absolute values on the secondary axis (on the right)

Between March 2020 and December 2021, a total of 26 225 COVID-19 confirmed cases and 773 related deaths (case fatality ratio = 3%) were reported, and 153 196 tests were performed, which corresponds to a positivity rate of 17.1%. The time series analysis shows a classic small lag between the confirmed cases and the deaths curves. A peak occurring around the months of May, June, and July each year of the study period for both confirmed cases and deaths was observed. The peaks of the confirmed cases and deaths time series coincided with the introduction and the spreading of SARS-CoV-2 variants in the country (Fig. 1). The time series of the positivity rate and number of tests followed almost the same trend as for the incidence (Fig. 2). During the months of May, June, and July, 14 564 (55.5%) confirmed cases and 460 (59.5%) deaths were reported with a case fatality ratio of 3.2%. During this period, 54 751 (35.7%) tests were performed with a positivity rate of 26.6%. During the other months, the number of reported confirmed cases and deaths was 11 661 (44.5%) and 313 (40.5%) respectively, with a case fatality ratio of 2.7%. The number of tests performed was 98 445 (64.3%) for a positivity rate of 11.9%.

### Spatial description of the epidemic

**Fig. 3:**
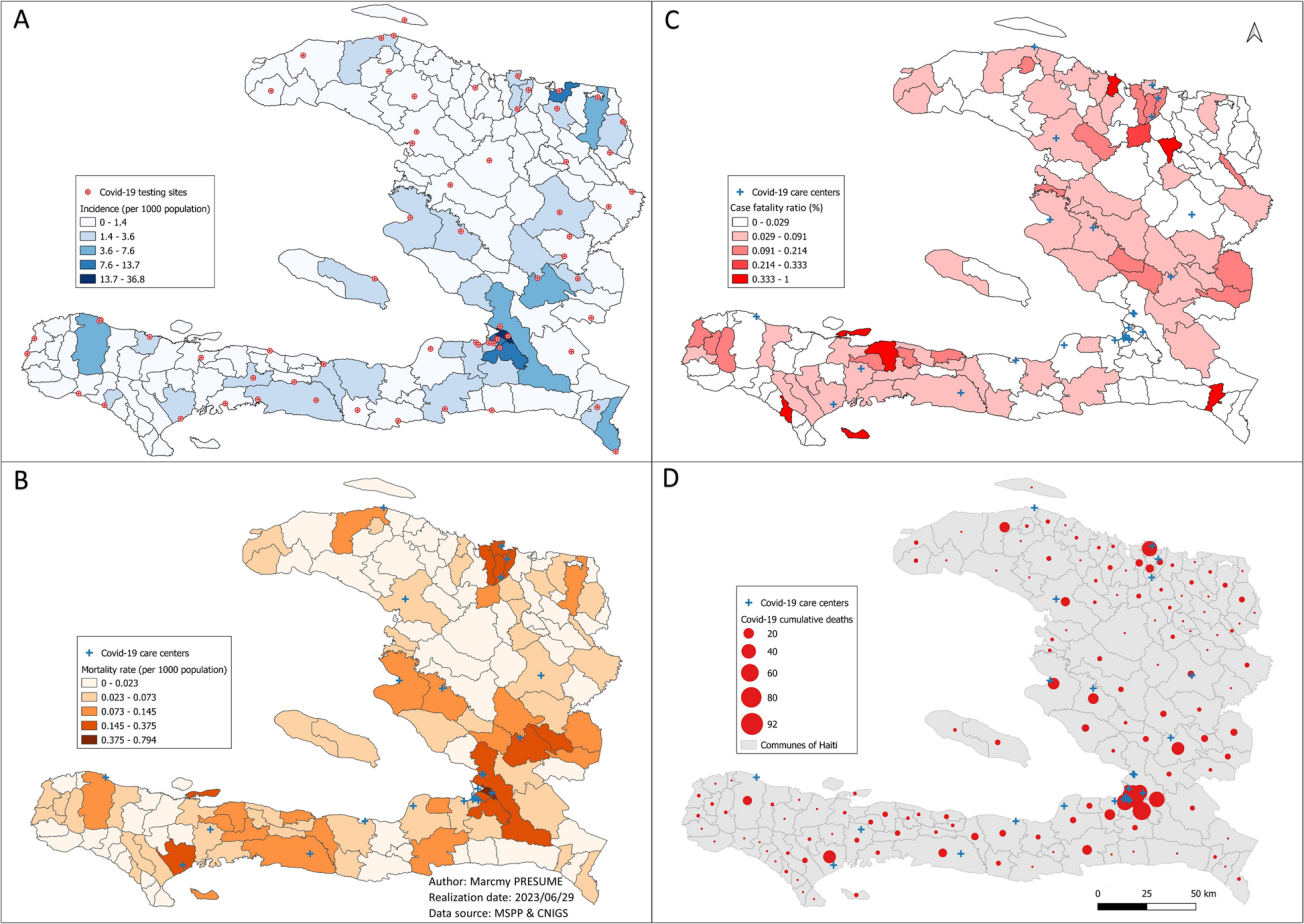
Spatial description of COVID-19 cumulative incidence and mortality rates, Haiti, March 2020-December2021. **A** Spatial distribution of the incidence in Haïti from March 2020 to December 2021; **B** Spatial distribution of the mortality rate (**C**) Spatial distribution of the lethality rate; **D** Spatial distribution of the covid-19 cumulative deaths

The spatial distribution of the cumulative incidence showed the same pattern as the mortality. We observed an aggregation of cases and deaths around the COVID-19 screening, and care centers, respectively. The case fatality ratio was higher in communes with lower incidence (Fig. 3).

**Fig. 4:**
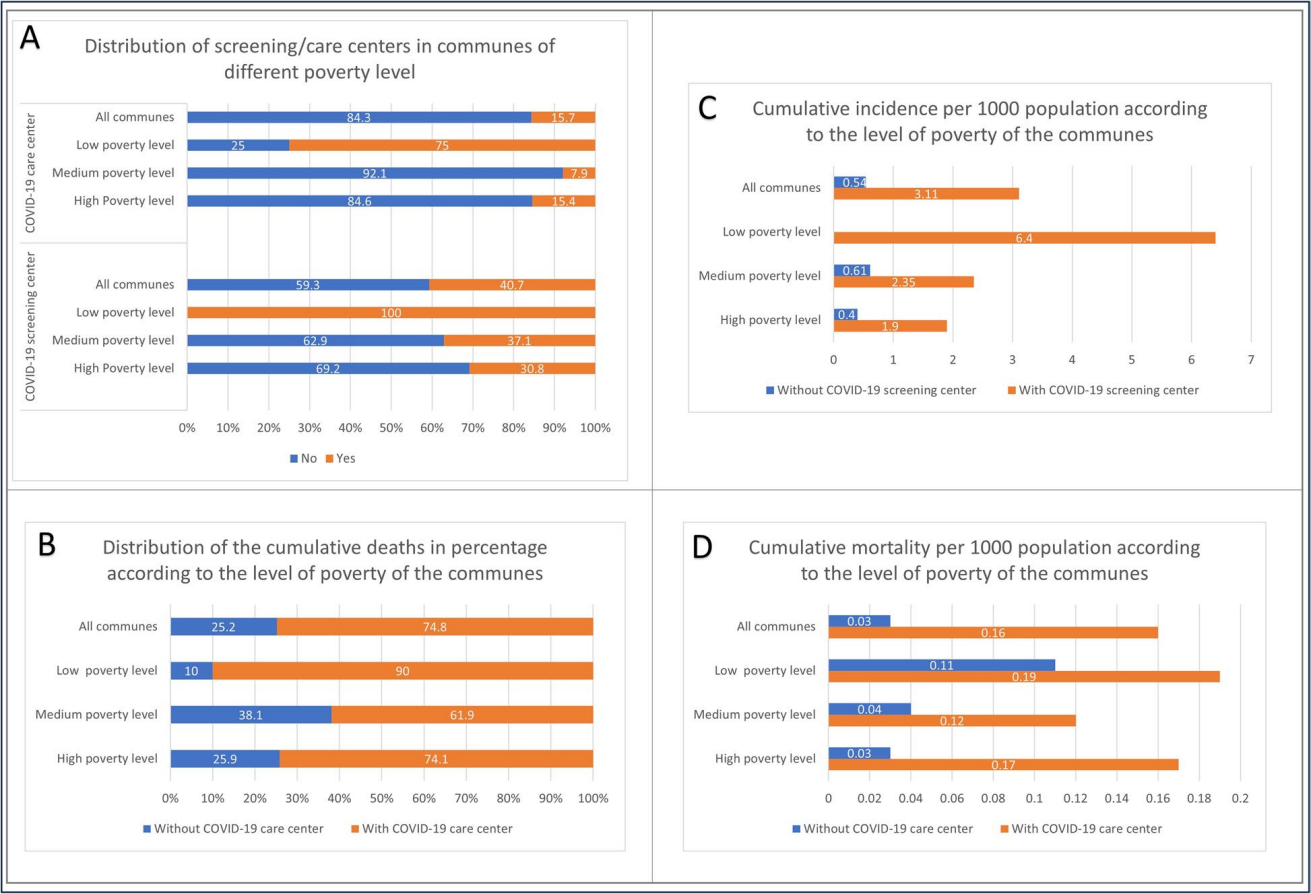
Incidence and mortality according to the poverty level of the communes, Haiti, March 2020-December 2021. **A** Distribution of screening/care centers in communes with different levels of poverty; **B** Distribution of the cumulative deaths in percentage according to the level of poverty of the communes; **C** Cumulative incidence per 1000 population according to the level of poverty of the communes; **D** Cumulative mortality per 1000 population according to the level of poverty of the communes

The cumulative incidence rate was 3.11 cases per 1000 population in the 57 (40.7%) communes with at least one COVID-19 screening center, and it was 0.54 cases per 100 population in the 83 (59.3%) communes without screening center. Only 22 (15.7%) communes had a COVID-19 care center and cumulated 74.8% of the total of deaths with a mortality rate of 0.16 deaths per 1000 population. The communes without COVID-19 care center were 118 (84.3%) and cumulated 25.2% of the total of deaths with a mortality rate of 0.03 deaths per 1000 population (Fig. 4).

The cumulative incidence and mortality rates were higher in communes with a low poverty level, independently of the presence of a COVID-19 screening and care center, respectively. Among the 12 communes with a low poverty level, all had at least one COVID-19 screening center and nine (75%) had at least one COVID-19 care center. The cumulative incidence in those communes was 6.40 cases per 1000 population and the mortality rate was 0.17 deaths per 1000 population. Among the 39 communes with a high poverty level, only 12 (30.8%) had a COVID-19 screening centers and six (15.4%) had a COVID-19 care center. The cumulative incidence in those communes was 0.87 cases per 1000 population and the mortality rate was 0.05 deaths per 1000 population (Fig. 4).

**Fig. 5:**
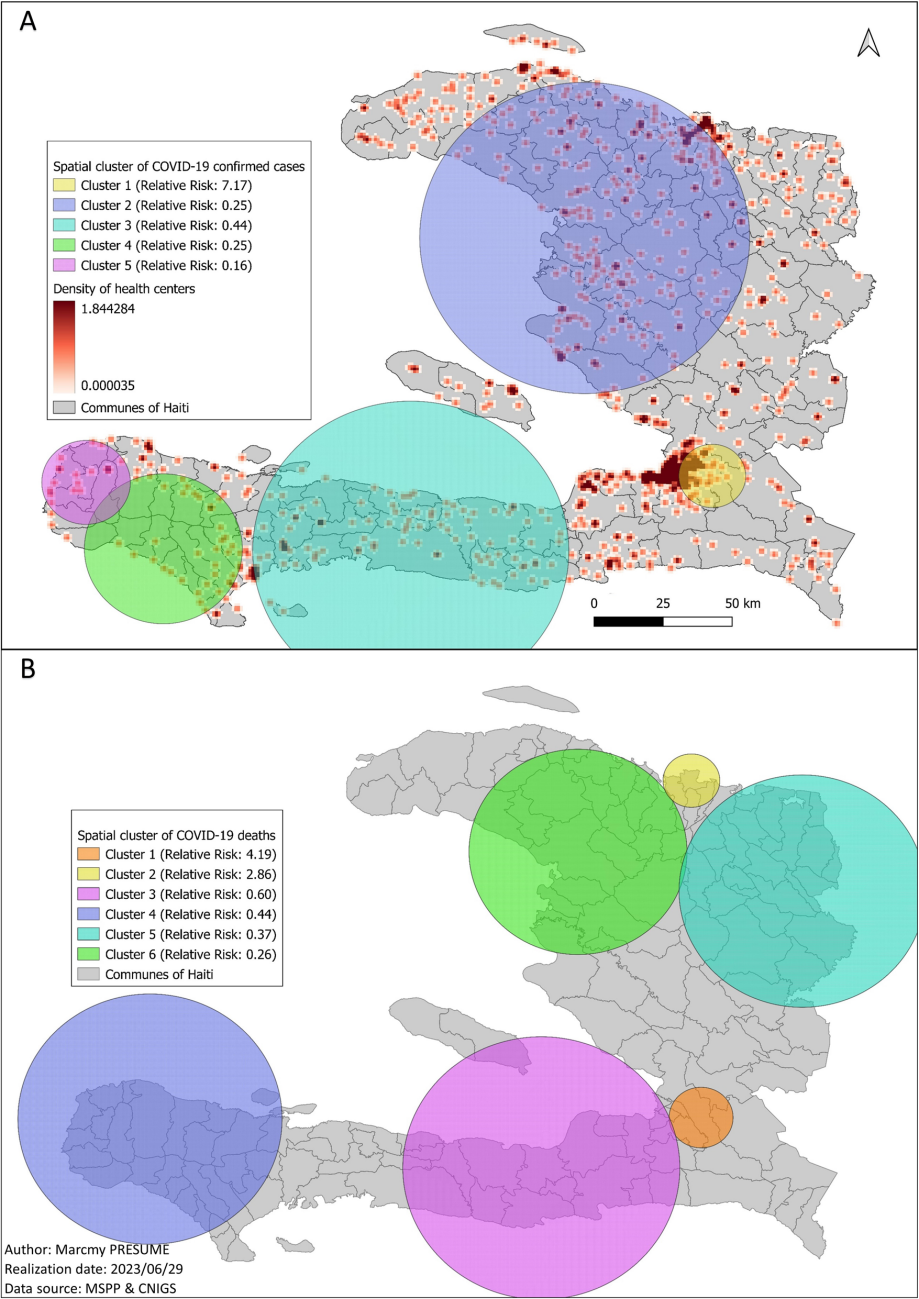
Description of the spatial clusters of COVID-19 cases and deaths, Haiti, March 2020-December 2021. **A** Description of the spatial clusters of covid-19 cases; **B** description of the spatial clusters of covid-19 deaths in Haiti from March 2020 to December 21

We found one high risk cluster and four low risk clusters for COVID-19 confirmed cases, and two high risk clusters and four low risk clusters for COVID-19 deaths. The high risk COVID-19 confirmed cases cluster (relative risk: 7.17, *p*-value < 0.001) and the higher risk COVID-19 deaths cluster (relative risk: 4.19, *p*-value < 0.001) were found around the metropolitan area, precisely in Delmas, Tabarre, Petion-Ville and Croix-des-Bouquets. Each of those related communes had on average two COVID-19 screening centers while 83 of 140 communes in the country had none. They each had 1.5 COVID-19 care centers on average while 118 of 140 communes had none. We observed an aggregation of health centers around those clusters with an average of 32.8 health centers per commune against 7.4 per commune on average for the whole country. The other high risk COVID-19 death cluster was located around the second main and most important city in the country, precisely in Cap-Haitien, Milot, Plaine-du-Nord and Quartier Morin. The risk relative for this cluster was 2.80 with a *p*-value < 0.001 (Fig. 5). The average number of health centers per commune in this cluster was 13 with 0.8 COVID-19 care centers.

### Socioeconomic factors associated with the incidence and mortality

We used multivariate Quasi-Poisson regressions adjusted on socioeconomic covariates, to find out socioeconomic factors associated with the spatial distribution of the incidence and mortality in Haiti. The two models explained 91% of the deviance of the COVID-19 incidence and 75% of the deviance of the mortality respectively. We found that the incidence and the mortality were negatively associated with the poverty level, with a standardized incidence ratio (SIR) of 0.23 (IC95%: 0.17, 0.33) and a standardized mortality ratio (SMR) of 0.35 (IC95%: 0.22, 0.55) for the high poverty level; and a SIR of 0.36 (IC95%: 0.29, 0.44) and a SMR of 0.37 (IC95%: 0.27, 0.50) for the moderate poverty level. The distance from the centroid of the commune to the nearest COVID-19 screening or care center were also negatively associated with the incidence (SIR: 0.97; IC95%: 0.95, 0.99) and the mortality (SMR: 0.98; IC95%: 0.97, 0.99), respectively. Having more than three healthcare workers per 1000 population in the commune was positively associated with both the incidence (SIR: 3.31; IC95%: 2.50, 3.93) and the mortality (SMR: 2.73; IC95%: 2.03, 3.66). The proportion of adults was associated with the mortality only (SMR: 1582.19; IC95%: 3.38, 739,934.30) (Table 1).

**Table 1:** Socioeconomic factors associated with the incidence and mortality in Haiti, March 2020-December2021

	**Incidence** *Deviance: 91%; n=140*		**Mortality** *Deviance: 75%; n=140*	
	**SIR**	**CI 95%**	**SMR**	**CI 95%**
Proportion of males < = 50%	1 (ref.)		1 (ref.)	
Proportion of males > 50%	1.00	(0.62, 1.60)	1.01	(0.59, 1.73)
Proportion of adults	204.34	(0.89, 47,050.47)	1582.19	(3.38, 739,934.30)
Household density	0.66	(0.30, 1.47)	0.86	(0.36, 2.08)
Urbanization rate < 0.5	1 (ref.)		1 (ref.)	
Urbanization rate > = 0.5	31.26	(0.51, 1897.84)	10.84	(0.09, 1336.63)
Poverty Level				
** Low**	1 (ref.)		1 (ref.)	
** Moderate**	0.36	(0.29, 0.44)	0.37	(0.27, 0.50)
** High**	0.23	(0.17, 0.33)	0.35	(0.22, 0.55)
Number of healthcare workers				
** < = 3 per 1000 population**	1 (ref.)		1 (ref.)	
** > 3 per 1000 population**	3.31	(2.50, 3.93)	2.73	(2.03, 3.66)
** Altitude (Kilometers)**	1.31	(0.77, 2.25)	0.70	(0.38, 1.31)
** Distance to the nearest COVID-19 screening center (Kilometers)**	0.97	(0.95, 0.99)	-	-
** Distance to the nearest COVID-19 care center (Kilometers)**	-	-	0.98	(0.97, 0.99)
Interaction of household density and urbanization rate:				
Household density * Urbanization rate < 0.5	1 (ref.)		1 (ref.)	
Household density * Urbanization rate > = 0.5	0.50	(0.21, 1.24)	0.58	(0.20, 1.67)

### Individual factors associated with the infection

Between March 2020 and December 2021, a total of 28 389 people presenting at least one symptom related to COVID-19 disease (mainly fever, cough, anosmia, dyspnea, vomiting, cold, and sore throat) were investigated. Among them, 4 442 (15.7%) had a positive COVID-19 antigen or PCR test. After adjusting on all other variables, male gender (OR adjusted: 1.11; IC95%: 1.01, 1.22), age with a progressive increase of the risk compared to youngers, and having Haitian nationality only (OR adjusted: 2.07; IC95%: 1.53, 2.82) where the main factors associated with the risk of being infected with the COVID-19 (Table 2).

**Table 2:** Factors associated with the risk of being infected with SARS-CoV-2, Haiti, March 2020-December 2021

	**n**	**N**	**%**		**OR **_**crude**_	**(CI 95%)**		**OR **_**adjusted**_	**(CI 95%)**
Sample	4 442	28 389	15.65		-	-		-	-
gender, male	2 463	28 389	8.68		1.24	(1.16, 1.32)		1.11	(1.01, 1.22)
Age									
** < = 19**	265	3 174	8.35		1 (ref.)	-		1 (ref.)	-
** 20–29**	853	6 024	14.16		1.81	(1.57, 2.10)		1.61	(1.33, 1.96)
** 30–39**	1 196	6 847	17.47		2.32	(2.02, 2.68)		1.94	(1.60, 2.36)
** 40–49**	792	4 674	16.94		2.24	(1.94, 2.60)		2.14	(1.75, 2.62)
** 50–59**	555	3 201	17.34		2.30	(1.97, 2.69)		2.20	(1.77, 2.72)
** 60–69**	407	2 439	16.69		2.20	(1.87, 2.59)		2.30	(1.84, 2.88)
** 70–79**	243	1 338	18.16		2.44	(2.02, 2.94)		2.64	(2.07, 3.38)
** 80 +**	131	692	18.93		2.56	(2.04, 3.21)		2.94	(2.20, 3.93)
** Having Haitian nationality only**	2 094	21 530	9.73		2.37	(1.77, 3.25)		2.07	(1.53, 2.82)
** Healthcare worker**	277	1 504	18.42		1.23	(1.07, 1.41)		1.11	(0.90, 1.36)
** Comorbidities**	768	5 258	14.61		0.91	(0.83, 0.98)		1.01	(0.90, 1.13)
Lifestyle									
** Smoking**	28	199	14.07		0.88	(0.58, 1.29)		1.26	(0.71, 2.22)
** Alcoholic**	22	218	10.09		0.60	(0.38, 0.92)		0.55	(0.28, 1.07)

## Discussion

This multidimensional study of the dynamics of the COVID-19 epidemic in Haiti from March 2020 to December 2021 highlights low COVID-19 incidence and mortality rates in the country globally, with a high degree of heterogeneity between communes. The cumulative incidence was six times higher in communes with at least one COVID-19 screening center, and the mortality five times higher in communes with at least one COVID-19 care center compared to those without. This pattern was confirmed in our multivariate spatial regression models where the distance to the nearest COVID-19 screening, and care center were negatively correlated to incidence and mortality, respectively, while the number of healthcare workers per population was positively correlated. These results suggest that there was a greater identification of cases and deaths around COVID-19 screening and care centers, respectively, highlighting an information bias.

We also found a negative correlation between the poverty level and the spatial distribution of incidence and mortality. The clusters at highest-risk of cases and deaths were in the same area, including Delmas, Petion-Ville, Tabarre and Croix-des-Bouquets. These communes are among the six richest ones in the country, according to a ranking based on the annual tax revenues of the communes [25]. In contrast, at the individual analysis level, those who have Haitian nationality only were more at risk compared to those who have at least one foreign nationality. Those who have at least one foreign nationality in Haiti have assets outside the country and have a higher standard of living than those who have Haitian nationality only. These results suggest an unequal distribution of COVID-19 screening and care centers in Haiti, explaining that the most vulnerable people have not been reached by the surveillance system. All the richest communes had at least one COVID-19 screening center and 75% of them had a COVID-19 care center while only 30.8% and 15.4% of the poorest communes had one, respectively. The communes forming the highest-risk clusters of confirmed cases and deaths belong to or are close to the metropolitan area which benefits from the highest level of accessibility to healthcare in the whole country. These results could also be explained by the refusal of the population to be tested and to use COVID-19 care centers despite symptoms, preferring to stay and die at home, as it has been reported elsewhere [26–28]. Studies on the effect of social deprivation on the dynamics of SARS-CoV-2 infection in France [29, 30], and in the United States [31] also reported that the underprivileged strata are more affected but less likely to be screened. However, in Haiti, distance to screening and care centers have certainly played a bigger role among this part of the population.

Our results showed that the positivity rate was three times higher than the 5% recommended by WHO [32]. This result supports the hypothesis of insufficient screening as the main driver why Haiti has recorded so few cases. Two seroprevalence studies in the period between August 2020 and July 2021 in Haiti found that the number of actual cases was significantly higher than that officially reported although these studies were carried out in health facilities and in areas with the highest accessibility to care [11, 12]. A population-based seroprevalence study conducted in the Dominican Republic from June to October 2021 found that 77.5% of the population over the age of 5 were previously infected with the virus, while the official reported cumulative incidence was less than 32 cases per 1000 population [7]. These results underscored the need to strengthen national surveillance systems low-income and middle-income countries, particularly for communities in remote rural areas [33].

The time series analysis of COVID-19 confirmed cases and deaths showed a peak that recurs during the months of May, June, and July. This period corresponds to a double climatic transition characterized by the beginning of the rainy period and the beginning of the hottest period of the year. However, the different peaks in the time series seem to be better explained by the emergence of different SARS-CoV-2 variants recorded in Haiti. A review of variants of concern and potential risk of new pandemic waves from December 2019 to December 2021 showed a rapid dissemination of new SARS-CoV-2 variants across the countries and reach a predominance of more than 80% [34].

At the individual level, male gender, and age with a progressive increase of the risk compared to youngers were associated with the infection. Since male gender and older age are risk factors to the severity of the COVID-19 disease [35], they are more likely to developpe symptoms and to be screened. These results are confirmed by other studies on risk factors for COVID-19 infection and severity [36, 37].

Sensitivity analysis showed overall stability in our results and consistent with the key message of our study. However, our study had some limitations. Firstly, our study had an ecological dimension which classically introduces an ecological inference bias. However, this bias was minimized by also taking results from individual data into account. Secondly, our study did not allow us to measure the gap between official confirmed cases and the actual number of cases at the population level, which would have allowed us to better model their determining factors. A population-based seroprevalence study would therefore be of great value to quantify the exact level of spread of the virus and their determining factors. Finally, undiagnosed community cases and their outcome remain unknown. It would be important not only to know how many they were, but also to assess their severity.

## Conclusion

Our study suggested that the number of COVID-19 cases and deaths were probably greatly underestimated in Haiti during the study period, mainly due to the very low number of tests performed and to a low coverage of COVID-19 screening and care centers. The poorest communes were less well served, adding inequality of access to social inequality. We recommend strengthening decentralized surveillance systems for epidemic-prone diseases, and setting up a real-time mortality surveillance system to better assess the impact of future health emergencies.

### Supplementary Information

Supplementary Material 1.

Supplementary Material 2.

## Data Availability

The individual datasets used and/or analyzed during the current study are not publicly available due to privacy policy but are available from the corresponding author on reasonable request. However, prior authorization from the Ministry of Health to access those data is mandatory. The other categories of dataset used and/or analyzed during the current study were collected from the Ministry of Health website (https://www.mspp.gouv.ht/page-covid-19/) and The WHO covid-19 detailed surveillance data dashboard (https://app.powerbi.com/view?r=eyJrIjoiYWRiZWVkNWUtNmM0Ni00MDAwLTljYWMtN2EwNTM3YjQzYmRmIiwidCI6ImY2MTBjMGI3LWJkMjQtNGIzOS04MTBiLTNkYzI4M%20GFmYjU5MCIsImMiOjh9%20&pageName=RapportSection637fbf136cce49d006f9). The resulting final datasets are available from the corresponding author on reasonable request.
