# Supplementary material for: Geographical coverage of SARS-CoV-2 screening and care centers in Haiti: what do national surveillance data tell us?
Source: BMC Public Health. 2024 Jun 28;24:1732. doi: 10.1186/s12889-024-19262-7 (PMC11214257; doi:10.1186/s12889-024-19262-7)
Supplement: Supplementary file 1 — Supplementary Material 1. [file 12889_2024_19262_MOESM1_ESM.pdf]

Comparison of incidence and mortality in Haiti with other countries

On the 653rd day after reporting officially their first COVID-19 confirmed case, Haiti registered a cumulative incidence rate of 2.32 confirmed cases per population and a mortality rate of 0.07 deaths per 1000 population. In comparison, the incidence and mortality rates were respectively 40.09 and 0.38 for Dominican Republic, 84.33 and 0.98 for United States, and 108.50 and 1.40 for France. After standardization on age and gender, the incidence did not significantly change with 3.02 for Haiti, 41.67 for Dominican Republic, 81.39 for Unites States, and 113.17 for France. But the standardized mortality became twice higher for Haiti (0.13‰), slightly higher for Dominican Republic (0.47‰), and around twice lower for United States (0.59‰) and France (0.58‰). Also, we observed that the standardized incidence was greater as the number of tests per population was high (table S1).

Table S1. cumulative incidence, mortality, and number of tests per (‰population), 653rd day after the first case

**Countries**	**Standardized incidence (‰)**	**Standardized mortality (‰)**	**Number of tests (‰)**
Haiti	3.02	0.13	13.55
Dominican Republic	41.67	0.47	109.58
United States	81.39	0.59	1 817.75
France	113.17	0.58	2 359.30

Additional file 1: Comparison of incidence and mortality in Haiti with other countries.
