# Supplementary material for: Geographical coverage of SARS-CoV-2 screening and care centers in Haiti: what do national surveillance data tell us?
Source: BMC Public Health. 2024 Jun 28;24:1732. doi: 10.1186/s12889-024-19262-7 (PMC11214257; doi:10.1186/s12889-024-19262-7)
Supplement: Supplementary file 2 — Supplementary Material 2. [file 12889_2024_19262_MOESM2_ESM.pdf]

Sensitivity analysis

We conducted sensitivity analysis for the produced models to assess their robustness and the stability of the results. Regarding the analysis of individual data, we analyzed separately data recorded during the months of May, June, and July of 2020 and 2021 corresponding to the two major peaks of the epidemic for the study period, and the reste of the sample. Secondly, we discarded the data from the COVID-19 high risk cluster to analyze the rest of the sample. Thirdly, we discarded the data from the metropolitan area to analyze the rest of the sample. We found an overall stability of our results. Only male gender was not at risk anymore in the sub-sample outside the two peaks period and in the sub-sample outside the metropolitan area (Table S2).

Table S2. Comparison of the results of the multivariate logistic model of the whole and sub-samples

	**Whole sample**	**the two peaks period**	**Outside the two peaks period**	**Outside the cluster zone**	**Outside the Metrop. area ***
gender, male	1.11 (1.01, 1.22)	1.23 (1.07, 1.43)	1.03 (0.91, 1.16)	1.11 (1.00, 1.22)	1.09 (0.99, 1.21)
Age					
<=19	Ref.	Ref.	Ref.	Ref.	Ref.
20-29	1.61 (1.33, 1.96)	1.57 (1.15, 2.15)	1.75 (1.36, 2.26)	1.74 (1.41, 2.16)	1.70 (1.37, 2.10)
30-39	1.94 (1.60, 2.36)	1.87 (1.38, 2.54)	2.08 (1.62, 2.68)	2.09 (1.70, 2.59)	2.06 (1.67, 2.53)
40-49	2.14 (1.75, 2.62)	1.99 (1.45, 2.73)	2.32 (1.79, 3.02)	2.34 (1.87, 2.91)	2.27 (1.83, 2.83)
50-59	2.2 (1.77, 2.72)	1.91 (1.37, 2.68)	2.39 (1.81, 3.16)	2.54 (2.02, 3.21)	2.34 (1.86, 2.95)
60-69	2.3 (1.84, 2.88)	2.22 (1.56, 3.15)	2.43 (1.81, 3.25)	2.69 (2.11, 3.43)	2.62 (2.06, 3.34)
70-79	2.64 (2.07, 3.38)	2.41 (1.63, 3.56)	2.77 (2.01, 3.81)	3.02 (2.32, 3.93)	2.83 (2.17, 3.69)
80+	2.94 (2.20, 3.93)	2.39 (1.46, 3.90)	3.57 (2.47, 5.16)	3.64 (2.67, 4.96)	3.47 (2.54, 4.72)
Have Haitian nationality only	2.07 (1.53, 2.82)	2.02 (1.24, 3.29)	1.99 (1.33, 2.97)	2.13 (1.51, 3.00)	1.88 (1.35, 2.61)

Healthcare worker	1.11 (0.90, 1.36)	1.27 (0.93, 1.74)	1.02 (0.76, 1.36)	1.16 (0.92, 1.45)	1.06 (0.84, 1.34)
Comorbidities	1.01 (0.90, 1.13)	0.95 (0.79, 1.14)	1.06 (0.92, 1.23)	0.94 (0.82, 1.06)	0.96 (0.84, 1.09)
Lifestyle					
Smoking	1.26 (0.71, 2.22)	1.23 (0.45, 3.34)	1.41 (0.69, 2.86)	1.30 (0.70, 2.40)	1.34 (0.72, 2.49)
Alcoholic	0.55 (0.28, 1.07)	0.90 (0.33, 2.40)	0.40 (0.16, 1.00)	0.55 (0.26, 1.14)	0.52 (0.25, 1.07)

** Metropolitan area*

Regarding the GAM model of the incidence, we removed and added successively all covariates in order to figure out how they affected the quality of the model. All covariates contributed to the quality of the model. We then analyzed separately the communes with at least one COVID-19 screening center, and the communes without a COVID-19 screening center. The poverty level and the number of healthcare workers per population remained associated and kept the sense of their association in the sub-samples. The distance to the nearest COVID-19 screening center was not associated anymore with the incidence in the sub-sample with COVID-19 screening center, but remained associated in the sub-sample without COVID-19 screening center. The altitude level became associated in the sub-samples with a negative association in the model with COVID-19 screening centers, and a positive association in the model without screening center (table S3).

Table S3. GAM model of the incidence - Comparison of the whole and sub-samples

	**Whole sample**	**With COVID-19 screening center**	**Without COVID-19 screening center**
	Deviance: 91%; n=140	*Deviance: 94.2%; n=57*	*Deviance: 81.9%; n=83*
Proportion of males <= 50%	1 (ref.)	1 (ref.)	1 (ref.)
Proportion of males > 50%	1 (0.62, 1.60)	0.79 (0.39, 1.59)	1.21 (0.87, 1.69)

Proportion of adults	204.34 (0.89, 47050.47)	0.37 (0.00, 652.04)	0.03 (0.00, 2.03)
Household density	0.66 (0.30, 1.47)	0.47 (0.16, 1.39)	0.90 (0.51, 1.61)
Urbanization rate < 0.5	1 (ref.)	1 (ref.)	1 (ref.)
Urbanization rate >= 0.5	31.26 (0.51, 1897.84)	5.46 (0.02, 1897.18)	88.68 (0.73, 10755.31)
Poverty Level			
Low	1 (ref.)	1 (ref.)	-
Moderate	0.36 (0.29, 0.44)	0.36 (0.28, 0.47)	1 (ref.)
High	0.23 (0.17, 0.33)	0.48 (0.30, 0.77)	0.60 (0.40, 0.88)
Number of healthcare workers			
<= 3 per 1000 population	1 (ref.)	1 (ref.)	1 (ref.)
> 3 per 1000 population	3.31 (2.50, 3.93)	1.51 (1.03, 2.23)	3.45 (2.36, 5.04)
Altitude (Kilometers)	1.31 (0.77, 2.25)	0.15 (0.06, 0.40)	2.26 (1.21, 4.21)
Distance to the nearest COVID-19 screening center (Kilometers)	0.97 (0.95, 0.99)	1.01 (0.98, 1.05)	0.97 (0.96, 0.99)
Interaction of household density and urbanization rate:			
Household density * Urbanization rate < 0.5	1 (ref.)		
Household density * Urbanization rate >= 0.5	0.5 (0.21, 1.24)	0.68 (0.19, 2.43)	0.44 (0.15, 1.29)

Regarding the GAM model of the mortality, we removed and added successively all covariates in order to figure out how they affected the quality of the model. All covariates contributed to the

quality of the model. We then analyzed separately the communes with at least one COVID-19 car center, and the those without a COVID-19 care center. Only the number of healthcare workers per population remained significantly and positively associated with the mortality in the sub-samples. The proportion of adults, the poverty level, and the distance to the nearest COVID-19 care center were not associated anymore to the incidence in the sub-samples (table S4).

Table S4. GAM model of the mortality - Comparison of the whole and sub-samples

	**Whole sample**	**With COVID-19 care center**	**Without COVID-19 care center**
	Deviance: 75%; n=140	*Deviance: 60.6%; n=22*	*Deviance: 50.5%; n=118*
Proportion of males <= 50%	1 (ref.)	1 (ref.)	1 (ref.)
Proportion of males > 50%	1.01 (0.59, 1.73)	0.95 (0.07, 12.49)	1.24 (0.70, 2.19)
Proportion of adults	1582.19 (3.38, 739934.30)	52.73 (0.00, 823400068.10)	41.52 (0.02, 74893.54)
Household density	0.86 (0.36, 2.08)	0.71 (0.01, 40.10)	0.79 (0.32, 1.96)
Urbanization rate < 0.5	1 (ref.)	1 (ref.)	1 (ref.)
Urbanization rate >= 0.5	10.84 (0.09, 1336.63)	1.24 (0.00, 2393024849.35)	6.03 (0.01, 2429.65)
Poverty Level			
Low	1 (ref.)	1 (ref.)	1 (ref.)
Moderate	0.37 (0.27, 0.50)	0.52 (0.19, 1.44)	0.74 (0.34, 1.60)
High	0.35 (0.22, 0.55)	0.55 (0.12, 2.48)	2.12 (0.74, 6.03)
Number of healthcare workers			

<= 3 per 1000 population	1 (ref.)	1 (ref.)	1 (ref.)
> 3 per 1000 population	2.73 (2.03, 3.66)	3.77 (1.56, 9.11)	2.05 (1.26, 3.31)
Altitude (Kilometers)	0.7 (0.38, 1.31)	0.44 (0.06, 3.25)	0.75 (0.27, 2.06)
Distance to the nearest COVID-19 care center (Kilometers)	0.98 (0.97, 0.99)	1.03 (0.94, 1.12)	0.99 (0.97, 1.00)
Interaction of household density and urbanization rate:			
Household density * Urbanization rate < 0.5	1 (ref.)		
Household density * Urbanization rate >= 0.5	0.58 (0.20, 1.67)	0.83 (0.01, 125.08)	0.99 (0.97, 1.00)

The sensitivity analysis supports the main conclusion of the study. In the logistic regression model, our results did not significantly change across the sub-samples. In the GAM models, all covariates contributed to the quality of the models. Except in the sample without COVID-19 screening center, the distance to the nearest COVID-19 screening or care center were not associated with the incidence or the mortality anymore in the sub-samples. Even if they were not statistically significant, their related SIR and SMR were greater than one (risk factor) in the samples with COVID-19 screening or care centers, and less than one (protective factor) in the sub-sample without COVID-19 screening or care center like in the whole sample. This supports the conclusion on an information bias, we don't have too much information about what appended in the communes without COVID-19 screening or care center.

Additional file 2: Sensitivity analysis performed for the produced models.
